# Reusable nanosilver-coated magnetic particles for ultrasensitive SERS-based detection of malachite green in water samples

**DOI:** 10.1038/srep22870

**Published:** 2016-03-11

**Authors:** Dan Song, Rong Yang, Chongwen Wang, Rui Xiao, Feng Long

**Affiliations:** 1 School of Environment and Natural Resources, Renmin University of China, 100872, Beijing, China; 2 Beijing Institute of Radiation Medicine, Beijing 100850, China

## Abstract

A novel nanosilver-deposited silica-coated Fe_3_O_4_ magnetic particle (Fe_3_O_4_@SiO_2_@Ag) with uniform size, good SERS activity and magnetic responsiveness was synthesized using amination polymer. The Fe_3_O_4_@SiO_2_@Ag magnetic particles have been successfully applied for ultrasensitive SERS detection of malachite green (MG) in water samples. The mechanism is that MG can be adsorbed on the silver surface of nanosilver-coated magnetic particles via one nitrogen atom, and the Raman signal intensity of MG is significantly enhanced by the nanosilver layer formed on the magnetic particles. The developed sensing system exhibited a sensitive response to MG in the range of 10 fM to 100 μM with a low limit of detection (LOD) 2 fM under optimal conditions. The LOD was several orders of magnitude lower than those of other methods. This SERS-based sensor showed good reproducibility and stability for MG detection. The silver-coated magnetic particles could easily be regenerated as SERS substrates only using low pH solution for multiple sensing events. The recovery of MG added to several water samples at different concentrations ranged from 90% to 110%. The proposed method facilitates the ultrasensitive analysis of dyes to satisfy the high demand for ensuring the safety of water sources.

Malachite green (MG) is a popular cationic triphenylmethane dye and has widely been used in the silk, dye, leather and textile industry^1^. Despite its prohibited use in aquaculture in many countries, it is widely utilized as a fungicide and parasiticide because of its high efficiency and low cost^1^,^2^,^3^. Unfortunately, MG is resistant to biodegradation because of its complicated and stable chemical structure (Figure S1)^4^, and is now a matter of concern because of its genotoxicity and carcinogenicity^5^,^6^. The ultrasensitive detection of MG has thus become important because this toxin can bioaccumulate in aquatic life before entering the food chain. This urgent global requirement is evident through the frequent occurrence of the incidents resulting from MG residues^7^. For instance, the British Food Standards Agency (FSA) found the banned MG in organic salmon sold at a leading supermarket in 2005, and China recently faced a variety of problems caused by MG residues^8^. Traditional analytical methods for MG include high-performance liquid chromatography, gas chromatography-mass spectrometry, and liquid chromatography-tandem mass spectrometry^9^,^10^. Although these methods allow measurements with high accuracy and sensitivity, they are expensive, complicated, and time consuming and require professional expertise. Hence, they are unsuitable for on-site or field applications.

To address the aforementioned issues, many MG-sensitive sensors based on electrochemistry, bioassay, and nanomaterials have been reported^11^,^12^. Although these approaches have made great contributions toward MG detection, most of them exhibit features that limit their practical applications, such as poor limit of detection (LOD), cross-sensitivity toward other small molecules, and the sophisticated synthesis of materials. Therefore, new methods must be developed to overcome these limitations. Surface-enhanced Raman Scattering (SERS), attributed to the combination of electromagnetic and chemical charge transfer mechanisms, demonstrates great potential for the ultrasensitive detection (ppb or less) of trace targets through the extraction of the molecular fingerprint information^13^. Several SERS-based nanosensors have been developed for MG detection based on the interaction of MG molecules with SERS active metal nanostructures^14^,^15^. Extensive effort has also been devoted to the design and fabrication of highly homogenous topologies and reproducible SERS-active nanostructure substrates, which requires advanced instruments and multi-step fabrication processes. Most of these sensors have an LOD at nM grade. However, the residues of MG in environmental samples and food usually have low concentration (<pM). Therefore, the development of simple, cost-effective, and ultrasensitive methods for the rapid determination of MG must be prioritized for the benefit of the environment and human health.

The major obstacle in the detection of MG is the regeneration and subsequent reuse of MG amalgamated materials after their exposure. Previously, MG has been demonstrated to have the strongest adsorption capability on the nanomaterial surfaces at pH 4–7, including silver particles^16^,^17^,^18^,^19^. However, they have little adsorption ability under lower pH conditions^19^. The Fe_3_O_4_@Ag magnetic particles with relatively high chemical stabilities facilitate acid treatment processes, thus enabling an additional functionality of surface regeneration through the acid removal of adsorbed MG.

The silver-coated magnetic particles are commonly synthesized with the help of an interlayer of silica shell^20^,^21^. The silica shell is used to prevent the aggregation of the magnetic particles and provide functional groups for further modification. In previous reports, the Ag^+^ ions were adsorbed on the surface of the Fe_3_O_4_@SiO_2_ by physical adsorption, and then reduced and deposited directly on the surface of Fe_3_O_4_@SiO_2_^20^. Ag particles with uniform size and coating state can hardly be obtained due to the further nucleation during the “growth” stage of the reduced reaction^21^. Furthermore, the ferromagnetic property of the magnetic cores may cause the final composite magnetic particles to aggregate severely^22^. To address these issues, we applied the “seed-mediated growth” strategy to produce nanosilver shell on the surface of magnetic particles. The “seed-mediated growth” method is a universal approach to prepare metal particles through adding new atoms onto the existing nuclei^23^. The uniform AuNP seeds on the surface of magnetic particles could allow the homogeneous growth of Ag layer^24^. In this study, the 3~5 nm Au NPs on the surface of Fe_3_O_4_@SiO_2_ act as nucleation sites for the deposition of the nanosilver shell. The prepared silver-coated magnetic particles had several advantages such as continuous silver shell, high SERS activity, good dispersity and strong magnetic responsiveness. The proposed SERS-active nanosilver-coated magnetic particles were for the first time used for the ultrasensitive detection of MG, which could be fully regenerated for application in multiple sensing events.

## Results

### Characterization of Fe_3_O_4_@SiO_2_@Ag magnetic particles

**Figure 1:**
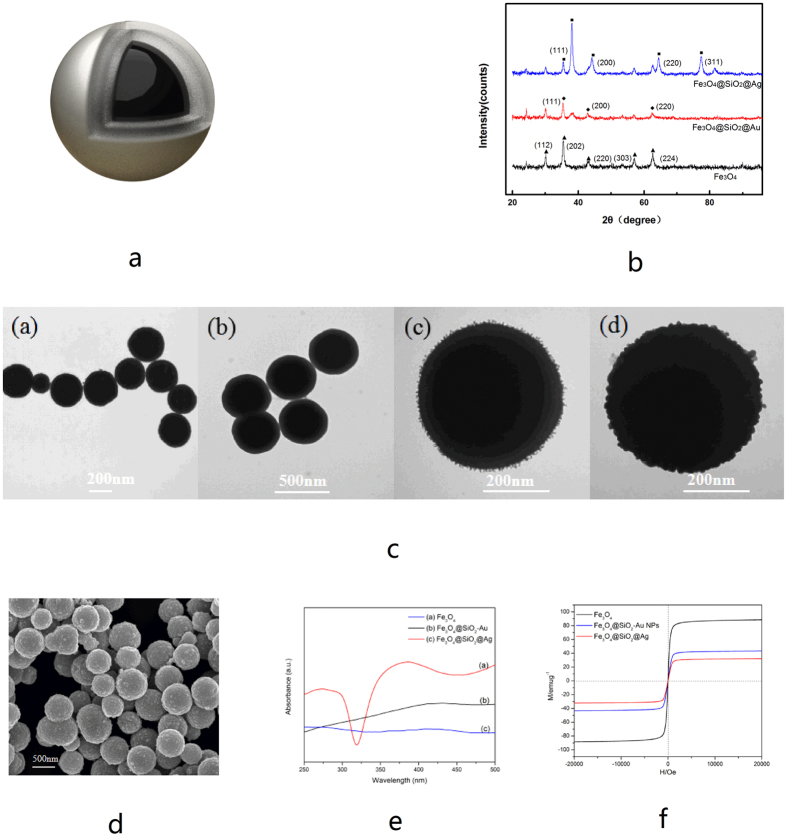
Characterization of Fe_3_O_4_@SiO_2_@Ag magnetic particles. (**a**) Schematic of Fe_3_O_4_@SiO_2_@Ag. (**b**) Typical XRD patterns of Fe_3_O_4_, Fe_3_O_4_@SiO_2_-Au seed, and Fe_3_O_4_@SiO_2_@Ag (**c**). TEM photos of Fe_3_O_4_ (**a**), Fe_3_O_4_@SiO_2_ (**b**), Fe_3_O_4_@SiO_2_-Au seed (**c**), and Fe_3_O_4_@SiO_2_@Ag (**d**). (**d**) SEM photo of Fe_3_O_4_@SiO_2_@Ag. (**e**) UV-vis spectra of Fe_3_O_4_, Fe_3_O_4_@ SiO_2_-Au seed, and Fe_3_O_4_@SiO_2_@Ag. (**f**) Magnetic hysteresis curves of Fe_3_O_4_, Fe_3_O_4_@ SiO_2_-Au seed, and Fe_3_O_4_@ SiO_2_@Ag at 300 K.

**Figure 2:**
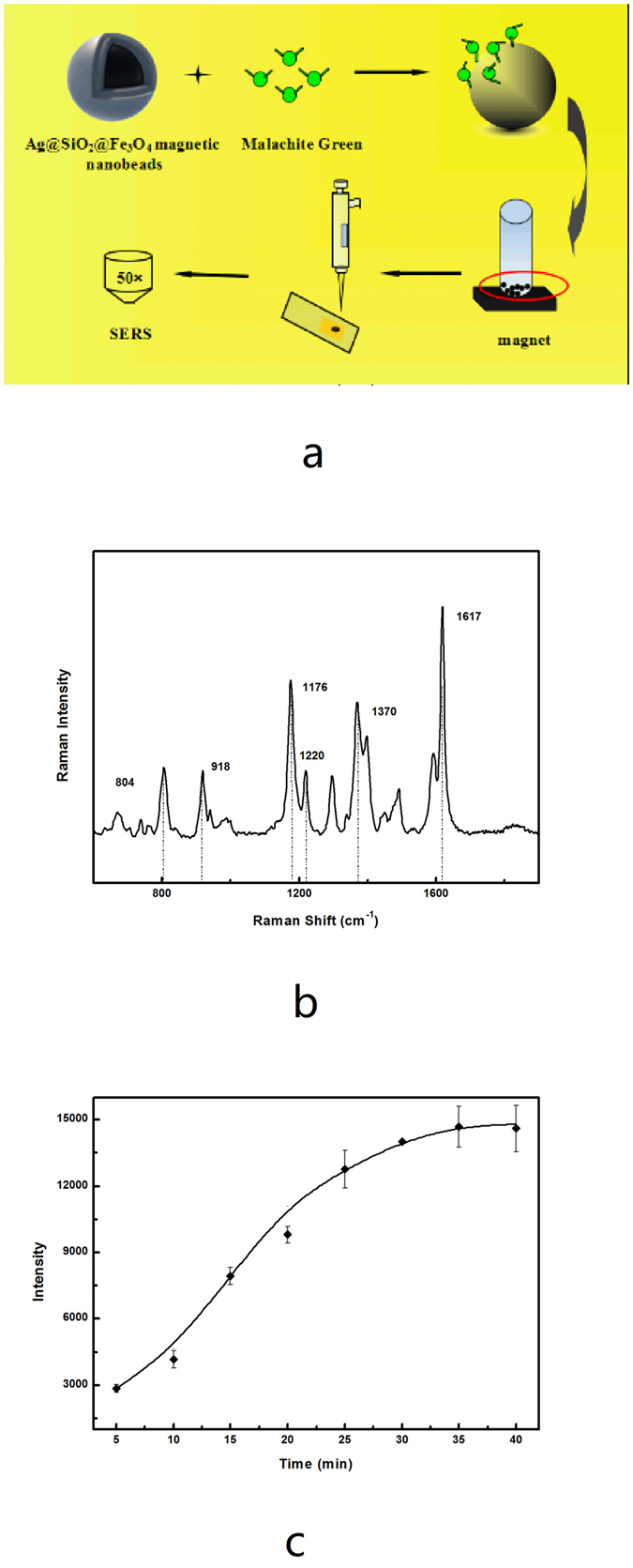
(**a**) SERS detection mechanism of MG using Fe_3_O_4_@SiO_2_@Ag magnetic particles. (**b**) Typical SERS detection spectra of 10^−3^ mol/L MG on magnetic particles measured on an inVia confocal Raman microscope with 633 nm laser, 1mW laser power, 10s exposure time, and one accumulation time. (**c**) Adsorption kinetics of MG on magnetic particles. The error bars represent standard deviation from eight measurements.

The schematic of Fe_3_O_4_@SiO_2_@Ag magnetic particles fabricated according to the above process is shown in Fig. 1a. Powder X-ray diffraction (XRD) was employed to verify the crystal structure and phase purity of the main synthetic product (Fig. 1b). The black curve in Fig. 1b shows the typical XRD pattern of the Fe_3_O_4_ particles. The diffraction peaks at 30°, 37.1°, 43°, 53.4°, 56.9°, and 62.5° correspond to the (112), (202), (220), (024), (303), and (224) planes of the cubic inverse spinel Fe_3_O_4_, respectively, all of which could be indexed to the cubic structure of Fe_3_O_4_ (JCPDS No.75-1609)^25^. The XRD pattern of the Fe_3_O_4_-SiO_2_-Au particle is characterized by three additional strong peaks positioned at the 2θ values of 38.2°, 44.3° and 64.5°, which correspond to the (111), (200), and (220) crystal planes of the cubic phase Au (JCPDS No.04-0784), respectively^26^. The relatively high intensity of the Au diffraction peaks proved the dense adsorption of 3~5 nm Au seeds on the Fe_3_O_4_@SiO_2_ surface (red curve in Fig. 1b). The deposition of silver can be proven from the XRD data shown as the blue curve in Fig. 1b. Three XRD peaks were clearly observed at the 2θ values of 38.1°, 44.3°, 64.4°, and 77.3° because of the reflections of the (111), (200), (220), and (311) crystalline planes of cubic Ag, respectively^27^,^28^. The characteristic peaks for Au and Ag were too close to distinguish^26^. The average size of Ag particles deduced from Scherrer’s formula is ~30 nm for the sample corresponding to Fig. 2c^ ^26. These results proved that nanosilver layer has been successfully grown on the surface of magnetic particles. The TEM (Fig. 1c) and SEM (Fig. 1d) image demonstrates the presence of numerous bumps on the surface of the magnetic particles, which greatly increased the surface area of the magnetic particles. This structure is beneficial for the increasing adsorption of MG and the enhancement of SERS signals^29^. SEM image showed that the as-prepared magnetic particles were spherical with a mean diameter of 645 ± 40 nm (Fig. 1d)

The UV-visible absorption spectra of Fe_3_O_4_, Fe_3_O_4_@SiO_2_-Au seed, and Fe_3_O_4_@SiO_2_@Ag, are shown in Fig. 1e. Curve (a) denotes the typical UV-visible spectra of a bare Fe_3_O_4_ particle, similar to that in the reference^30^. The Fe_3_O_4_@SiO_2_-Au seed particles did not show any obvious UV-visible absorption in the range of 250 nm~500 nm (curve (b) of Fig. 1e). After the formation of the nanosilver shell, a broad plasmonic resonance peak appeared at around 382 nm because of the Mie plasmon resonance from silver particles (curve (c) of Fig. 1e)^31^. This result further confirmed the adhesion of nanosilver layer on the Fe_3_O_4_@Ag surface.

The magnetic properties of the products were examined using a superconducting quantum interference device magnetometer (SQUID, MPMSXL-7) at 300 K. As shown in Fig. 1f, the saturation magnetization (MS) values of Fe_3_O_4_, Fe_3_O_4_@SiO_2_-Au seed, and Fe_3_O_4_@SiO_2_@Ag were found to be 88.7, 41.6, and 32.5 mu/g, respectively. The MS values showed a decreasing trend after SiO_2_-coating, Au seed-absorbing, and Ag shell-forming. Such decrease was mainly due to the mass effect of silica and silver and partly due to diamagnetic shielding^24^. However, the Fe_3_O_4_@SiO_2_@Ag particles could be magnetically concentrated and readily picked up using a small magnet. All of the curves nearly intersected with the origin, thus indicating all of the three products were in a superparamagnetic state at room temperature^32^. In the practical magnetic separation test, Fe_3_O_4_@SiO_2_@Ag could be completely separated from the solution within 10 s with the application of a magnet. Such a short separation time reflects the potential of these magnetic particles when used in rapid enrichment of target analytes.

### SERS detection mechanism and adsorption kinetics of MG

The SERS detection process of MG using Fe_3_O_4_@SiO_2_@Ag magnetic particles is shown in Fig. 2a. As described in the experimental section, the MG solution of different concentrations was mixed with magnetic particles for a certain time. Previous research has demonstrated MG adsorption on silver surfaces via only one nitrogen atom in a tilted upright configuration, as verified by the strongly enhanced in-plane vibration modes of MG^18^. After the adsorption of MG on the surface of the Fe_3_O_4_@SiO_2_@Ag magnetic particles in the present study, the mixture was separated with a magnet. Then, 2 μL magnetic particles was dropped on the gold film on a glass plate and then dried for the Raman measurements (Figure S2).

The typical SERS spectrum of MG on magnetic particles is shown in Fig. 2b. The most prominent peaks of MG appeared at wave numbers of 1,176, 1,370 and 1,617 cm^−1^ because of ring C-H in-plane bending, N-phenyl stretching, and ring C-C stretching^33^, respectively. Significant electromagnetic enhancements were induced by the resonance of localized surface plasmon on the surface of the nanosilver coated on magnetic particles. A relatively large effect of SERS was observed because of the molecular resonance of MG having a maximum absorption at 620 nm wavelength, which is very close to that of the incident laser (633 nm)^34^. Although the Raman characteristic peak at 1,173 cm^−1^ was mostly used to distinguish the MG molecules because of the low Raman vibration signal from the SERS-active surface^15^,^35^,^36^, a highly sensitive Raman peak at 1,617 cm^−1^ was selected for the trace detection of MG molecules in the environmental samples^14^,^37^.

Adsorption kinetics of MG is shown in Fig. 2c. MG adsorption at 10^−4^ mol/L and pH 4.5 increased by rising contact time, eventually slowed down and finally reached equilibrium after 30 min (Figure S3). At the initial stage, the adsorption of MG was rapid because the more active sites on the surface of Ag-coated magnetic particles exist, which led to acceleration of MG mass transfer. However, the active site amount of Ag coated magnetic particle were limited and decreased with the adsorption of MG, and the adsorption amount of MG reached a platform after 30 min. Therefore, the optimum adsorption time of 30 min was selected for quantitative detection of MG in the following experiments.

### Effect of pH on adsorption of MG on silver-coated magnetic particles

**Figure 3:**
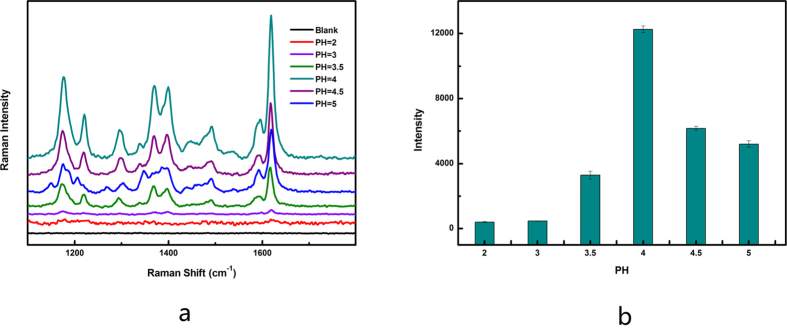
Effect of pH on the adsorption of MG on silver-coated Magnetic particles. (**a**) SERS spectra of MG by varying its pH from 2 to 5, the concentration of MG is 0.1 mM. (**b**) Raman peak position at 1617 cm^−1^ under different pH. The error bars represent standard deviation from eight measurements.

Several studies have demonstrated that the adsorption of MG on nanomaterials is greatly affected by the pH of the solutions, thus making the surface adsorption via a single dimethylamino group insufficiently stable^14^,^37^. To evaluate the effect of pH on the adsorption of MG on silver-coated magnetic particles, 100 μL of MG solution (0.1 mM) with different pH values was mixed with 50 μL magnetic particles (1 mg/mL). After 30 min, the mixture was separated with a magnet, and 2 μL magnetic particle solution was dropped on the gold film for SERS detection. As shown in Fig. 3, pH demonstrated a significant effect on the adsorption between the MG molecule and the silver film on the magnetic particles. At a pH value lower than 4, the SERS signal increased with the increasing pH of the MG solution. At a pH value higher than 4, the SERS signal slightly decreased. When the pH of the solution was lower than 2, few MG molecules could be adsorbed on the surface of silver-coated magnetic particles. These results are almost consistent with those of previous reports^37^. The effect of pH may be attributed to two factors. First, as the pH of the solution (pH < 4) decreased, the positive charge originating from the absorption of the hydrogen ions (H^+^) onto the silver surface produced a repulsive force that hindered from MG adsorption^37^,^38^. Second, the excess H^+^ ions may compete with the dye cations for the adsorption sites of the silver-coated magnetic particles^37^,^38^.

To verify that the SERS signal change did result from the effect of pH in the solution on the amount of MG adsorbed on the surface of the magnetic particles, we also studied the effect of pH on the SERS signal of MG itself. The 2 μL of MG solution (0.1 mM) with different pH values was directly dropped on the gold film, and the Raman signal was detected. The experimental results showed that the pH of the solution did not affect the SERS signal of MG itself (Figure S4). Therefore, the decrease in the SERS signal of the MG solution with decreasing pH resulted from the decreasing amount of MG adsorbed on the surface of the magnetic particles.

### Ultrasensitive SERS-based detection of MG using nanosilver-coated magnetic particles

**Figure 4:**
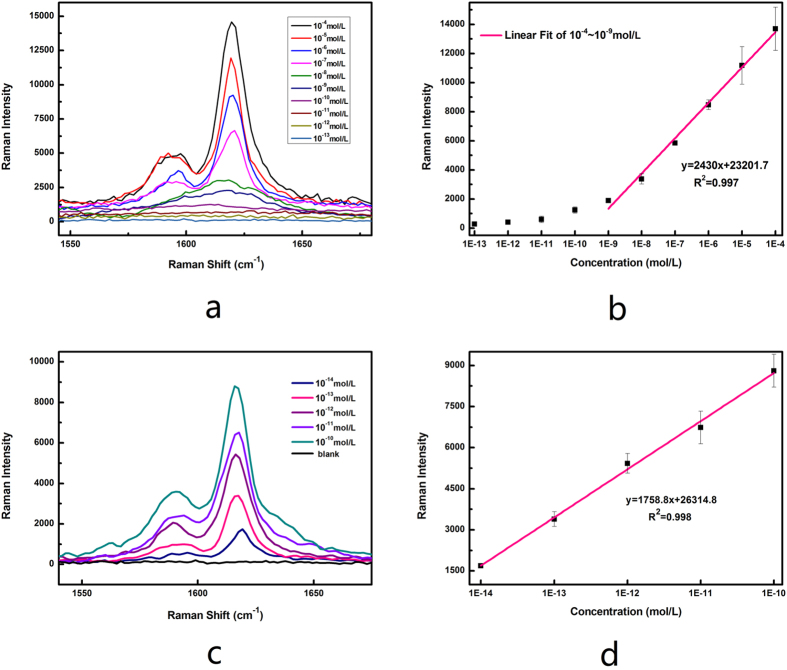
SERS detection of MG using silver-coated magnetic particles. (**a**) SERS spectra of MG of different concentration ranging from 10^−4^ mol/L to 10^−14^ mol/L adsorbed on magnetic particles. Detection condition: Laser wavelength 633 nm, accumulation time 1, exposure time 10s, and laser power 1 mW. (**b**) 1617 cm^−1^ band intensities versus the concentration of MG, and line fitness was performed ranged from 10^−4^ mol/L to 10^−14^ mol/L MG. (**c**) SERS spectra of low concentration of MG ranging from 10^−10^ to 10^−14^ mol/L. Detection condition: Laser wavelength 633 nm, accumulation times 5, exposure time 10s, and laser power 1 mW. (**d**) Plot of 1617 cm^−1^ band intensities versus the low concentration of MG at five accumulation times. The error bars represent standard deviation from eight measurements.

The typical SERS spectra of the SERS active systems under different MG concentrations are presented in Fig. 4a. An increase in SERS intensity was observed as the MG concentration increased from 10^−14^ mol/L to 10^−4^ mol/L. The increasing trend of the SERS intensity with MG concentration is summarized further in the inset of Fig. 4b. The intense SERS peak at 1617 cm^−1^ in the spectra was used as a calibration band. When the concentration of MG exceeded 1 nM, the linear equation of the calibration curve is determined as y = 2430x + 23201.7 (x > 1 nM) with a correlation coefficient R^2^ = 0.997, where y is the SERS intensity and x is the logarithm of MG concentration.

The detection of low MG concentration is important because MG residue comes in trace amounts in drinking water or wastewater treat plant effluent^6^,^14^. However, the intensity was so low that it was difficult to apply for the quantitative detection of MG (Fig. 4a). The change in the exposure time, the laser power and accumulation times should increase Raman intensity. The optimization results are illustrated in the supporting materials (Figure S5–7). Although the increase in the laser power and exposure time had no obvious effect on the enhancement of the Raman intensity of MG at low concentrations, the accumulation time had an important influence on the improvement of the Raman intensity. With the increase of the accumulation time, the MG solution of 10^−14^ mol/L which had no Raman signal under previous conditions, gradually increased the Raman intensity at 1617 cm^−1^. The five accumulation times was been selected in this study to save time. Figure 4c demonstrates that MG at low concentrations can also be detected under new conditions because the linearity correlation coefficient is 0.998. In this case, the LOD was determined to be 2 × 10^−15^ mol/L by using 3σ/*S* calculation parameter (average standard deviation of measurements (σ) and the slope of the dose-response (*S*) fitting curve). The ultrahigh sensitivity of MG detection can contribute to the following reasons. First, the direct adsorption of MG on silver layer of magnetic particle can effectively increase SERS enhancement factor. Second, the presence of numerous bumps on the surface of the magnetic particles greatly increases the SERS active surface area, which is beneficial for the increasing adsorption of MG and the enhancement of SERS signals. Third, increasing accumulation times can improve the sensitivity of MG detection as described above.

### Detection of real samples using SERS based nanosensors

To evaluate the potential matrix effect of real environmental water samples on the performance of the proposed method, several samples including lab tap water, bottled water, and secondary sedimentation effluent water were tested. These samples were spiked with MG from the MG stock solution at 1 μM, 10 nM, 100 pM, and 1 pM concentrations. The results were summarized in Table S1. The recovery of all the measured samples ranged from 90% to 110%, and the parallel tests showed that the coefficient of variation was <10% (n = 4). The results showed that the proposed method was sensitive and accurate in the successful determination of ultralow levels of MG in the real water samples.

### Regeneration and stability of Fe_3_O_4_@SiO_2_@Ag magnetic particles

**Figure 5:**
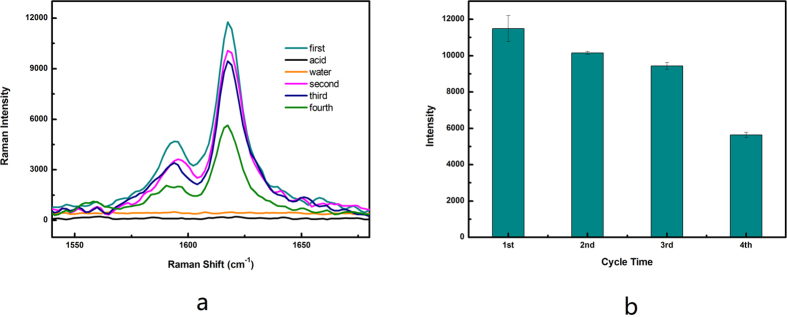
Regeneration performance of Fe_3_O_4_@SiO_2_@Ag magnetic particles. (**a**) SERS spectra of MG (10^−5^ mol/L) on magnetic particles with different regeneration cycles. Black line: the SERS signal curve of Fe_3_O_4_@SiO_2_@Ag magnetic particles after regeneration using HCl solution (pH = 2); Orange line: the SERS signal curve of Fe_3_O_4_@SiO_2_@Ag magnetic particles washed by water after acid treatment. (**b**) Raman peak position at 1617 cm^−1^ at different regeneration cycles. The error bars represent standard deviation from eight measurements.

The regeneration ability of magnetic particles is essential for practical applications, as it can reduce the overall cost for the target detection. According to the results presented above, we noted that the amount of MG adsorbed on the surface of the magnetic particles was low under a low pH solution. We assumed that the MG adsorbed on the magnetic particles should be released through washing with a low pH solution because the positive ions (H^+^) compete with MG on the surface of magnetic particles. To validate this assumption, 100 μL MG solution (10^−5^ mol/L) was mixed with 1 mg magnetic particles for 30 min. Following the separation and washing of the magnetic particles, 2 μL magnetic particles were applied for SERS detection. The other magnetic particles were washed by HCl solution (pH = 2) thrice to remove the adsorbed MG molecules. The SERS signal intensity of magnetic particles washed by HCl solution is very low, indicating the complete removal of the MG adsorbed on magnetic particles (Fig. 5). After the washing of the magnetic particles with water, the 100 μL MG solution (10^−5^ mol/L) was added to test whether the magnetic particles could be reused as SERS substrates. The result is shown in Fig. 5. The adsorption capacity decreased for each new cycle, while the adsorption capacity remained at 80% after three cycles. Therefore, the magentic particles could be reused as SERS substrate at least thrice using a low pH solution for regeneration. When the magnetic particles were in dark stored in a refrigerator at 4 °C for 30 days, the decrease in the SERS signal of 10^−4^ mol/L MG and 10^−7^ mol/L MG was 6.05% and 4.46% (Figure S8), respectively, indicating that the proposed sensor had enough stability for MG measurement.

## Discussions

In summary, a novel nanosilver-coated magnetic particle with uniform size, good SERS activity and magnetic responsiveness were synthesized by using polymer PAH. The nanosilver-coated magnetic particles were successfully applied for ultrasensitive SERS detection of MG in water samples. This SERS-active sensing platform exhibits several distinctive advantages that are not available from conventional dye sensing systems. First, MG could be detected with ultrahigh sensitivity (2 fM) using the silver-coated magnetic particles. Second, the silver-coated magnetic particles could be readily regenerated as SERS substrate using low pH acid to perform multiple sensing events, which makes it the defined protocol a low-cost technique. In additional, this proposed method was successfully employed to determine MG in several spiked environmental samples without obvious matrix effect. Although we only demonstrated the detection of MG, we envisioned that the demonstrated strategy had great potential in the ultrasensitive analysis and effective removal of other dyes and small molecules, which satisfied the high demand for ensuring the safety of water sources.

## Methods

### Materials and reagents

Ferric chloride hexahydrate (FeCl_3_-6H_2_O), silver nitrate (AgNO_3_), sodium acetate, adenine, malachite green chloride (Product of Switzerland), polyethylene glycol (MW 3400), PEG6000, and poly(allylamine hydrochloride) (PAH, MW 70 kDa) were purchased from Sigma-Aldrich and used as received. Ammonia solution (28.0–30.0 wt.%) was obtained from Sanchun Pure Chemical Company. Other chemicals, unless specified, were of reagent grade, and highly purified water was used throughout the experiments. A stock solution of MG (10^−3^ mol/L) was prepared by dissolving the appropriate amount of malachite green chloride in highly purified water. An array of strong neodymium bar magnet in size of 3 mm in diameter was purchased from AIM Magnet Co.Ltd. TMR Heating ThermoMixers and KQ-250DB numerical controlled ultrasonic cleaner were also be used in the study.

### Fabrication of Ag@SiO_2_@Fe_3_O_4_ magnetic particles

The Fe_3_O_4_ magnetic particles (400 nm) were firstly synthesized through a modified solvothermal reaction^39^. Typically, 1.35 g of FeCl_3_·6H_2_O was dissolved in 40 mL of ethylene glycol under magnetic stirring for 30 min. Subsequently, 2.7 g of NaAc and 1 g of PEG 6000 were added to this solution and stirred until the reactants were fully dissolved. Then, the mixture was transferred into a Teflon-lined autoclave (50 mL capacity) and heated at 200 °C for 12 h. The products were collected with the help of a magnet, followed by washing with deionized water and ethanol three times, respectively. The final product was dried under vacuum at 60 °C for 5 h. To prepare SiO_2_@Fe_3_O_4_ magnetic particles, the 0.1 g as-prepared Fe_3_O_4_ particles were dispersed in a deionized water-ammonia-ethanol mixture solution (5:4:5, v/v) by sonication for 15 min^40^. 100 μL of tetraethoxysilane (TEOS) was slowly dropped into the above solution by vigorous sonication. The mixture was kept at room temperature for 45 min. The product was dried in a vacuum oven at 60 °C for 5 h, followed by washing with deionized water and ethanol.

The surface amination of Fe_3_O_4_@SiO_2_ magnetic particles was achieved through a PAH self-assembly process under sonication. Typically, 0.25g PAH was dissolved in de-ionized water (50 mL) by ultrasonication for 10 min, and 0.1 g as-prepared Fe_3_O_4_@SiO_2_ particles were added under sonication for another 20 min. PAH gradually self-assembled on the Fe_3_O_4_@SiO_2_ particles. Then, the resulted Fe_3_O_4_@SiO_2_@PAH particles were magnetically separated and rinsed five times with deionized water. Next, 3~5 nm gold-seed was synthesized as described in previous method with some modification^41^. Briefly, 20 mL of HAuCl_4_ (5 mM) and 20 mL of trisodium citrate (5mM) were added to 360 mL de-ionized water and vigorously stirred. Afterwards, 10 mL of freshly-prepared NaBH_4_ (0.1 M) was added and the solution color changed from colorless to orange. Then, the solution was stirred for 4 h at RT and the resulting spherical gold particles were about 3~5 nm in diameter. PAH-modified SiO_2_@Fe_3_O_4_ particles and AuNPs were mixed to form Fe_3_O_4_@SiO_2_@PAH-AuNPs through the electrostatic interaction. After sonication for 1 h, the mixture was separated by a magnet and washed three times with deionized water. For obtaining Fe_3_O_4_@SiO_2_@Ag, 10 mg Fe_3_O_4_@SiO_2_-Au NPs was dispersed in 100 mL AgNO_3_ solution (0.25 mM, containing 0.2 wt% PVP). The excessive amount of 37% formaldehyde (150 μL) and 25% ammonia solution (300 μL) were added in sequence. The Fe_3_O_4_@SiO_2_@Ag magnetic particles were obtained within 2 min under sonication at 30 °C. The products were magnetically separated and washed five times with deionized water to remove the excess PVP.

### SERS detection of MG

For the quantitative analysis of MG concentration, a standard MG solution from 10^−3^ mol/L to 10^−14^ mol/L was prepared with MG stock solution. For the SERS measurement, 50 μL of MG solution of different concentrations was mixed with 100 μL Fe_3_O_4_@SiO_2_@Ag magnetic particles in an EP tube, which was shaken in a TMR Heating ThermoMixer (1,200 rpm) at room temperature for 30 min to prevent particle deposition. Afterward, the mixtures were separated with a magnet, and the supernatant was removed. With the aid of an ultrasonic cleaner, the magnetic particles were washed by purified water thrice. Then, 10 μL of purified water was added to the EP tube, and the magnetic particles were thoroughly mixed again. Subsequently, 2 μL of the sample solution was dropped on a 50 nm gold film deposited on a glass plate. After drying at room temperature, the SERS spectra of the MG adsorbed on the magnetic particles were detected by Raman spectroscopy. To achieve the ultrasensitive detection of MG, several experimental conditions, such as laser power, exposure time, and accumulation times, were optimized. To study the influence of initial pH on the adsorption and detection of MG, the initial pH values of the solution were adjusted to 2, 3, 3.5, 4, 4.5 and 5 with addition of hydrochloric acid.

To evaluate the potential matrix effects of the environmental samples on MG detection, spiked samples of lab tap water, bottled water, and secondary sedimentation effluent (Qinghe Wastewater Plant, Beijing, China) were tested at concentrations of 1 μM, 10 nM, 100 pM, and 1 pM.

### Apparatus

SERS measurements were performed on an inVia confocal Raman microscope (British Renishaw Company) with 633 nm excitation laser, 50 × long distance objective, and 1,200 lines/mm gratings (note that the SERS signals of low MG concentration were obtained via accumulation that was repeated five times). The pH measurements were obtained with a pH meter (FE20-FiveEasy from METTLER TOLEDO). TEM images were obtained using Hitachi transmission electron microscope (H-7650B, Japan). Scanning electron microscopy (SEM) images were captured using an S-4800 scanning electron microscope (Hitachi, Japan). The absorbance measurements were carried out on Nanodrop 2000 (American Thermo Fisher Company). TMR Heating ThermoMixers and KQ-250DB numerically controlled ultrasonic cleaners were also used in the experiment.

## Additional Information

**How to cite this article**: Song, D. *et al.* Reusable nanosilver-coated magnetic particles for ultrasensitive SERS-based detection of malachite green in water samples *Sci. Rep.* **6**, 22870; doi: 10.1038/srep22870 (2016).

## Supplementary Material

Supporting Information

## References

[b1] Forgacs E., Cserháti T. & Oros G. Removal of synthetic dyes from wastewaters: A review. Environ. Int. 30, 953–971 (2004). 15196844 10.1016/j.envint.2004.02.001

[b2] Zhou X. J., Guo W. Q., Yang S. S., Zheng H. S. & Ren N. Q. Ultrasonic-assisted ozone oxidation process of triphenylmethane dye degradation: evidence for the promotion effects of ultrasonic on malachite green decolorization and degradation mechanism. Bioresour. Technol. 128, 827–830 (2013). 23199816 10.1016/j.biortech.2012.10.086

[b3] Oladoja N. & Aliu Y. Snail shell as coagulant aid in the alum precipitation of malachite green from aqua system. J. Hazard. Mater. 164, 1496–1502 (2009). 19013019 10.1016/j.jhazmat.2008.09.114

[b4] Khairud M., Muhammad D., Kooh R. R. & Linda B. L. Water remediation using low cost adsorbent walnut shell for removal of malachite green: Equilibrium, kinetics, thermodynamic and regeneration studies. J. Environ. Chem. Eng. 2, 1434–1444 (2014).

[b5] Culp S. J. *et al.* A. Carcinogenicity of malachite green chloride and leucomalachite green in B6C3F1 mice and F344 rats. Food Chem. Toxicol. 44, 1204–1212 (2006). 16554117 10.1016/j.fct.2006.01.016

[b6] Srivastava S., Sinha R. & Roy D. Toxicological effects of malachite green. Aquat. Toxicol. 66, 319–329 (2004). 15129773 10.1016/j.aquatox.2003.09.008

[b7] Lee S. Choi J., Chen L. & Park B. Fast and sensitive trace analysis of malachite green using a surface-enhanced Raman microfluidic sensor. Anal. Chim. Acta 590, 139–144 (2007). 17448337 10.1016/j.aca.2007.03.049

[b8] Zhang Y. *et al.* Development of chemiluminescent enzyme immunoassay for the determination of malachite green in seafood. Food Agr. Immun. 26, 204–217 (2015).

[b9] Halme E., Lindfors E. & Peltonen K. Determination of malachite green residues in rainbow trout muscle with liquid chromatography and liquid chromatography coupled with tandemmass spectrometry. Food Addit. Contam. 21, 641–648 (2004). 15370837 10.1080/02652030410001721457

[b10] Bueno M. J. M. *et al.* Determination of malachite green residues in fish using molecularly imprinted solid-phase extraction followed by liquid chromatography-linearion trap mass spectrometry. Anal. Chim. Acta 665, 47–54 (2010). 20381689 10.1016/j.aca.2010.03.001

[b11] Sahraei R., Farmany A. & Mortazavi. S. S. A nanosilver-based spectrophotometric method for determination of malachite green in surface water samples. Environ. Monit. Assess. 185, 5817–5822 (2013). 23203820 10.1007/s10661-012-2986-1

[b12] Mohammadifar E., Shemirani F., Majidi B. & Ezoddin M. Application of modified nano-γ-alumina as an efficient adsorbent for removing malachite green (MG) from aqueous solution. Desalin. Water Treat. 54, 758–768 (2015).

[b13] Nie S. & Emery S. R. Probing single molecules and single particles by surface-enhanced Raman scattering. Science. 275, 1102–1106 (1997). 9027306 10.1126/science.275.5303.1102

[b14] Sivashanmugan K. *et al.* Ag nanoclusters on ZnO nanodome array as hybrid SERS-active substrate for trace detection of malachite green. Sens. Actuators B 207, 430–436 (2015).

[b15] Han B., Choi N., Kim K. H., Lim D. W. & Choo. J. Application of silver-coated magnetic microspheres to a SERS-based optofluidic sensor. J. Phys. Chem. C. 115, 6290–6296 (2011).

[b16] Ghaedi M., Shojaeipour E., Ghaedi A. M. & Sahraei. R. Isotherm and kinetics study of malachite green adsorption onto copper nanowires loaded on activated carbon: Artificial neural network modeling and genetic algorithm optimization. Spectrochim. Acta Part A: Mol. Biomol. Spectro. 142, 135–149 (2015). 10.1016/j.saa.2015.01.086 25699703

[b17] Li D., Li D. W., Fossey J. S. & Long Y. T. Cyclic electroplating and stripping of silver on Au@SiO_2_ core/ shell particles for sensitive and recyclable substrate of surface-enhanced raman scattering. J. Mater. Chem. 20, 3688–3693 (2010).

[b18] Fischer D., Caseri W. R. & Hahner G. Orientation and electronic structure of ion exchanged dye molecules on mica: An X-ray absorption study. J. Colloid Interface Sci. 198, 337 (1998).

[b19] Onal Y. C., Basar A. C. & Ozdemir S. Investigation kinetics mechanisms of adsorption malachite green onto activated carbon. J. Hazard. Mater. 146, 194–203 (2007). 17194532 10.1016/j.jhazmat.2006.12.006

[b20] Hu H. B., Wang Z. H., Pan L., Zhao S. P. & Zhu S. Y. Ag-coated Fe3O4@SiO2 three-ply composite microspheres: synthesis, characterization, and application in detecting melamine with their surface-enhanced Raman scattering. J. Phys. Chem. C 114, 7738–7742 (2010).

[b21] Zhang *et al.* Modified *in situ* and self-catalytic growth method for fabrication of Ag-coated nanocomposites with tailorable optical properties. J. Nanopart. Res. 14, 1105 (2012).

[b22] An Q. *et al.* Fe_3_O_4_@carbon microsphere supported Ag–Au bimetallic nanocrystals with the enhanced catalytic activity and selectivity for the reduction of nitroaromatic compounds. J. Phys. Chem. C 116, 22432–22440 (2012).

[b23] Li J. M. *et al.* Poly(styrene-co-acrylic acid) core and silver particle/silica shell composite microspheres as high performance surface-enhanced Raman spectroscopy (SERS) substrate and molecular barcode label. J. Mater. Chem. 21, 5992–5998 (2011).

[b24] Wang *et al.* Polyethylenimine-interlayered silver-shell magnetic-core microspheres as multifunctional SERS substrates. J. Mater. Chem. C 3, 8684–8693 (2015).

[b25] Deng H. *et al.* Monodisperse magnetic single-crystal ferrite microspheres. Angewandte Chemie 117, 2842–2845 (2005). 10.1002/anie.200462551 15798982

[b26] Lou L. *et al.* Facile methods for synthesis of core-shell structured and heterostructured Fe_3_O_4_@Au nanocomposites. Appl. Surf. Sci. 258, 8521–8526 (2012).

[b27] He R., Qian X., Yin J. & Zhu Z. Preparation of polychrome silver particles in different solvents. J. Mater. Chem. 12, 3783–3786 (2002).

[b28] Shen J., Zhu Y., Yang X., Zong J. & Li C. Multifunctional Fe_3_O_4_@Ag/SiO_2_/Au core-shell microspheres as a novel SERS-activity label via long-range plasmon coupling. Langmuir. 29, 690–695 (2013). 23206276 10.1021/la304048v

[b29] Zhang R. *et al.* Chemical mapping of a single molecule by plasmon-enhanced Raman scattering. Nature. 498, 82–86 (2013). 23739426 10.1038/nature12151

[b30] Wheeler D. A., Adams S. A., López-Luke T., Torres-Castro A. & Zhang J. Z. Magnetic Fe_3_O_4_-Au core-shell nanostructures for surface enhanced Raman scattering. Annalen der Physik. 524, 670–679 (2012).

[b31] Sauerbeck C. *et al.* Shedding Light on the Growth of Gold Nanoshells. ACS Nano. 8, 3088–3096 (2014). 24552660 10.1021/nn500729r

[b32] Ge J., Zhang Q., Zhang T. & Yin Y. Core-satellite nanocomposite catalysts protected by a porous silica shell: controllable reactivity, high stability, and magnetic recyclability. Angew. Chem. Int. Ed. Engl. 47, 8924–8928 (2008). 18925600 10.1002/anie.200803968

[b33] Lai K. *et al.* Determination of chloramphenicol and crystal violet with surface enhanced Raman spectroscopy. Sens. Instrument. Food Qual. Safety. 5, 19–24 (2011).

[b34] Pieczonka N. P. & Aroca R. F. Single molecule analysis by surfaced enhanced Raman scattering. Chem. Soc. Reviews. 37, 946–954 (2008). 10.1039/b709739p 18443680

[b35] Tan E. Z., Yin P. G., You T. T., Wang H. & Guo L. Three dimensional design of large-scale TiO_2_ nanorods scaffold decorated by silver particles as SERS sensorfor ultrasensitive malachite green detection. ACS Appl. Mater. Interfaces 4, 3432–3437 (2012). 22708788 10.1021/am3004126

[b36] Upadhyayula V. K. Functionalized gold particle supported sensory mechanisms applied in detection of chemical and biological threat agents: A review. Analy. Chim. Acta 715, 1–18 (2012). 10.1016/j.aca.2011.12.008 22244163

[b37] Xie H. *et al.* Deciphering surface enhanced Raman scattering activity of goldnanoworms through optical correlations. J. Phys. Chem. C 115, 20515–20522 (2011).

[b38] Leng W. & Vikesland P. J. Nanoclustered gold honeycombs for surface-enhanced Raman scattering. Anal. Chem. 85, 1342–1349 (2013). 23210677 10.1021/ac301028w

[b39] Liu J. *et al.* Microwave absorption enhancement of multifunctional composite microspheres with spinel Fe_3_O_4_ cores and anatase TiO_2_ shells. Small. 8, 1214–1221 (2012). 22331748 10.1002/smll.201102245

[b40] Wang Y. *et al.* Magnetic-based silver composite microspheres with nanosheet-assembled shell for effective SERS substrate. J. Mater. Chem. C. 1, 2441–2447 (2013).

[b41] Fang Y., Guo S., Zhu C., Zhai Y. & Wang E. Self-assembly of cationic polyelectrolyte-functionalized graphene nanosheets and gold particles: A two-dimensional heterostructure for hydrogen peroxide sensing. Langmuir. 2010, 26, 11277–11282. 20232834 10.1021/la100575g

