# Supplementary material for: Reusable nanosilver-coated magnetic particles for ultrasensitive SERS-based detection of malachite green in water samples
Source: Sci Rep. 2016 Mar 11;6:22870. doi: 10.1038/srep22870 (PMC4786807; doi:10.1038/srep22870)
Supplement: Supporting Information [file srep22870-s1.doc]

**Supporting Materials for**

Reusable nanosilver-coated magnetic particles for ultrasensitive SERS-based detection of malachite green in water samples

*Dan Song^1^, Rong Yang^1^, Chongwen Wang^2^, Rui Xiao^2^, Feng Long^1*^*

^1^School of Environment and Natural Resources, Renmin University of China, 100872, Beijing,

^2^Beijing Institute of Radiation Medicine, Beijing 100850, China

**Figure S1.** Molecular structure of malachite green

**
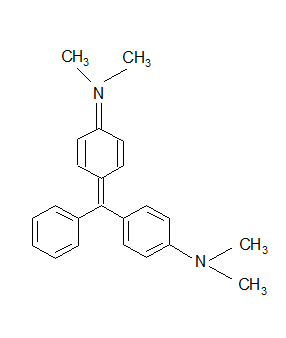
**

**Figure S2 Photo of SERS Raman detection (Red labeled circles were detection points)**

**
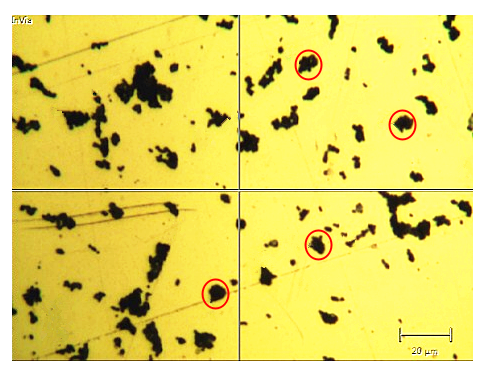
**

**Figure S3.** Time-dependent SERS spectra of MG (10^-4^ mol/L) on magnetic nanoparticles with the increasing reaction time (from 5 min to 40 min). Detection condition: Laser wavelength 633nm, accumulation times 2, exposure time 10s, and laser power 1 mW.

**Figure S4.** Effect of pH on MG’s Raman intensity, and pH are 1, 2, 3, 4, 5, 6, and 7 from 1 (a) to 7(g). Detection condition: Laser wavelength 633nm, accumulation times 2, exposure time 10s, and laser power 1 mW.

**Figure S5.** Effect of different exposure time in Raman intensity of MG (10^-14^mol/L). The exposure times changed from 5s to 30s. Detection condition: Laser wavelength 633nm, accumulation times 3, and laser power 1 mW.

**Figure S6.** Effect of laser power on Raman intensity of MG (10^-14^mol/L). The laser power changed from 0.1 mW to 10 mW. Detection condition: Laser wavelength 633nm, accumulation times 3, and exposure time 10s.

**Figure S7.** Effect of accumulation time on Raman intensity of MG (10^-14^mol/L). The accumulation times changed from 1 (a) to 9 (i). Detection condition: Laser wavelength 633nm, laser power 1 mW, and exposure time 10s.

**Figure S8.** Stability of the magnetic nanoparticles. (a) The Raman intensity of 10^-4^ mol/L MG using as-prepared magnetic nanoparticles and magnetic nanoparticles after 30 days storage in a refrigerator at 4 ºC, respectively. (b) The Raman intensity of 10^-7^ mol/L MG using as-prepared magnetic nanoparticles and magnetic nanoparticles after 30 days storage in a refrigerator at 4 ºC, respectively.

As-prepared MNP

Storage MNP

MG concentration: 10^-4^ mol/L

**(a)**

As-prepared MNP

Storage MNP

MG concentration: 10^-7^ mol/L

**(b)**

**Table S1. Detection results of water samples spiked with MG**.

| Sample | MG added | MG detected | Recovery(%) | C.V.(%) |
| --- | --- | --- | --- | --- |
| Lab Tap water | 1μM  10nM  100pM  1pM | 0.986μM  9.76nM  106pM  1.04pM | 98.6  97.6  106  104 | 4.27  3.14  1.78  1.32 |
| Bottle water | 1μM  10nM  100pM  1pM | 0.964μM  10.7nM  96.7pM  0.979pM | 96.4  107  96.7  97.9 | 4.51  5.58  2.23  1.41 |
| Seconary  Sedimentation  Effluent | 1μM  10nM  100pM  1pM | 1.02μM  9.93nM  103pM  1.017pM | 102  99.3  103  101.7 | 3.84  3.15  1.16  4.12 |

All samples were collected on 15^th^ of May, 2015, and the initial concentration of MG in them was undetectable. The reliability of data are expressed as coefficient of variation (CV, %; n = 4).
